# Robotic Partial Cystectomy for Urachal Adenocarcinoma: Surgical Technique with Cystoscopic Correlation

**DOI:** 10.1590/S1677-5538.IBJU.2025.0446

**Published:** 2025-09-10

**Authors:** Gabriel Chahade Sibanto Simões, Julio Calderón, Oliver Rojas Claros, Wladimir Alfer, Arie Carneiro

**Affiliations:** 1 Hospital Israelita Albert Einstein Serviço de Urologia São Paulo SP Brasil Serviço de Urologia, Hospital Israelita Albert Einstein, São Paulo, SP, Brasil

## Abstract

**Introduction and Objective:**

Urachal adenocarcinoma is a rare malignancy that typically arises from the bladder dome and is often diagnosed incidentally or during evaluation for hematuria (1, 2). Surgical excision remains the cornerstone of treatment (3, 4). This video demonstrates the robotic technique for partial cystectomy with en bloc urachal resection, combined with synchronized intraoperative cystoscopic imaging—an approach that has proven feasible and oncologically safe in selected cases (5-7).

**Materials and Methods:**

A 74-year-old hypertensive male presented with self-limited gross hematuria. Cystoscopy revealed a lesion at the bladder dome, and transurethral resection confirmed urachal adenocarcinoma. Staging chest and abdominal CT scans showed no evidence of metastasis. Robotic partial cystectomy with urachal excision was performed using a da Vinci platform and an auxiliary 5-mm port, in line with contemporary minimally invasive techniques, including single-port surgery (8). The urachus was dissected to the umbilicus, the bladder dome excised, and the bladder closed in two layers with barbed sutures. The specimen was retrieved through the umbilical incision following concomitant umbilectomy. Intraoperative cystoscopy provided simultaneous tumor visualization via picture-in-picture overlay.

**Results:**

Operative time was 45 minutes, with an estimated blood loss of 20 mL and no complications. The patient was discharged on postoperative day one with a Foley catheter, which was removed after seven days. Pathological analysis revealed moderately differentiated urachal adenocarcinoma with a mucinous component, detrusor and perineural invasion, and negative margins. At 6-month follow-up, the patient remained asymptomatic, continent, and recurrence-free on surveillance CT.

**Conclusion:**

Robotic partial cystectomy with urachal excision is a safe and effective treatment for localized urachal adenocarcinoma. Intraoperative cystoscopy enhances anatomical correlation and may contribute to oncologic safety.

**Available at:**VIDEO

## Data Availability

Not applicable
